# Novel Classification of Early-stage Systemic Hypertensive Changes in Human Retina Based on OCTA Measurement of Choriocapillaris

**DOI:** 10.1038/s41598-018-33580-y

**Published:** 2018-10-11

**Authors:** Kei Takayama, Hiroki Kaneko, Yasuki Ito, Keiko Kataoka, Takeshi Iwase, Tetsuhiro Yasuma, Toshiyuki Matsuura, Taichi Tsunekawa, Hideyuki Shimizu, Ayana Suzumura, Eimei Ra, Tomohiko Akahori, Hiroko Terasaki

**Affiliations:** 1 Department of Ophthalmology, National Defense Medical College, 3-2 Namiki, Tokorozawa, 359-8513 Japan; 2 Department of Ophthalmology, Nagoya University Graduate School of Medicine, 54 Tsurumai-cho, Showa-ku, 466-8550 Japan

## Abstract

The traditional classification of hypertensive retinopathy was based on the Keith–Wagener–Barker (KWB) grading, which is a subjective scaling system, and it is difficult to distinguish between the first and second grades. Retinal and choroidal vasculatures are affected by systemic hypertension, although retinal vasculature changes with age, axial length, intraocular pressure, and retinal diseases. It is necessary to establish a new objective method to assess hypertensive vascular changes. In the present study, we have examined the vasculature of the macular choriocapillaris in order to establish a new objective method to assess hypertensive vascular changes using optical coherence tomography angiography (OCTA). Choriocapillaris vessel density (VD), vessel length, and vessel diameter index in a 3 × 3 mm macular area were measured by OTCA in a total of 567 volunteers (361 healthy subjects and 206 subjects with systemic hypertension) who attended a basic health check-up. Ocular factors, systemic factors, and medications were evaluated. We detected significant differences in normative choriocapillaris vasculature between the left and right eyes in 53 healthy subjects and revealed correlations between age, intraocular pressure, axial length, and choriocapillaris vasculature in 308 healthy subjects. Normative foveal VD was correlated with age only and the efficiency was weak. The analysis of 206 right eyes (KWB grade 0, 159 eyes; grade 1, 35 eyes; and grade 2, 12 eyes) revealed that foveal VD was strongly correlated with KWB grade only (*P* < 0.001). This is the first report suggesting that OCTA for foveal choriocapillaris measurement by OCTA would might provide the advantage of evaluating be objective method for evaluating the progression of systemic hypertension.

## Introduction

Hypertensive retinopathy refers to a spectrum of retinal microvascular signs that develop in response to elevated blood pressure (BP)^1,2^ and does not imply a sequential temporal relationship per se. The traditional classification of hypertensive retinopathy was based on the Keith–Wagener–Barker (KWB) grading system^3,4^, which showed that the severity of retinopathy was an indicator of overall mortality in hypertensive patients and still used in worldwide^5,6^. The KWB scale remains the most widely cited grading system, but it has some limitations, such as the subjective scaling among clinicians and the difficulty in distinguishing between first and second grades^7,8^. Therefore, a more simplified classification that is relevant to current clinical practice is needed^9^, even if it is based on objective evaluation.

Retinal vasculature and choroidal vasculature are affected by systemic hypertension, and are also Retinal and choroidal vasculature is expected to change with age^10,11^, axial length (AL)^12,13^, intraocular pressure (IOP)^14,15^, and many diseases^16–24^. Such as diabetic retinopathy, retinal vascular occlusion, and epiretinal membrane disorders, making the estimation of systemic hypertensive vascular changes difficult. It can be challenging to notice early stage of retinopathy due to the systematic hypertensive and the symptoms can be mistaken with other retinal diseases. Therefore, further work to find more sensitive biomarkers of the systematic hypertensive in the eye is necessary. The choriocapillaris vasculature, which is small vessels with a lumen slightly larger than that of a typical capillary layer contains several blood vessels and major biological function is to supply oxygen and metabolites to the retinal pigment epithelium (RPE) and the outer neurosensory retina^25^, is decreased in the eyes of elderly individuals and those with systemic hypertension^26^, and few diseases such as macular degeneration^27^. The choriocapillaris^28,29^, pigments, and immune components^30^. It has and is arranged in a distinct layer limited to the inner portion of the choroid. The level of the choriocapillaris is so tightly arranged in the posterior pole that distinct capillary tubes are difficult to identify. A major biological function of the choriocapillaris is to supply oxygen and metabolites to the RPE and the outer neurosensory retina^25^, which have the highest metabolic demand of all biological tissues^31^, and are the only routes for metabolic exchange in the retina within the foveal avascular zone^32^.

Indocyanine green angiography (ICGA) has long been considered the gold standard for imaging the choroidal vasculature^33^. ICGA imaging is unable to individually evaluate blood flow or separate the choriocapillaris vasculature from the retinal and choroidal vasculature. Furthermore, ICGA is an invasive technique that requires intravenous dye injections, and it may cause mild or severe adverse reactions occasionally^34,35^. Optical coherence tomography angiography (OCTA) is a recently introduced state-of-the-art technique that can detect the movement of erythrocytes in the vascular network and can provide non-invasive imaging of the microvascular network without the use of dye injections^25,26,36^, so its application is not limited by contraindications to the dye. The ability of OCTA to measure depth enables the selective analysis of choriocapillaris vascularization in the retinal capillary plexus and choroidal vascularization. On the basis of the OCTA image produced by the analysis software, data on vessel density (VD) or vessel length (VL) can be automatically generated^37–39^, and the vessel diameter index (VDI), showing the mean vessel diameter, can be calculated as

$${\rm{VDI}}\,(\mu {\rm{m}})={\rm{VD}}\,({{\rm{mm}}}^{2}/{\mathrm{mm}}^{2})\times 1000/\mathrm{VL}\,({\mathrm{mm}/\mathrm{mm}}^{2}).$$

We hypothesized that the choriocapillaris vasculature is affected by the severity of systemic hypertension and can be objectively measured by OCTA. Therefore, in this study, we examined the choriocapillaris vasculature in eyes with and without systemic hypertension and compared the outcomes with those that were based on the KWB classification.

## Results

### Subjects’ characteristics

A total of 567 volunteers (361 healthy subjects and 206 subjects with systemic hypertension, all Japanese) were enrolled in the study. Three-hundred and eight eyes of 308 healthy subjects (147 men and 161 women, Table 1) were examined to detect whether any ocular/systemic factor affects choriocapillaris vasculature. Subsequently, 106 eyes from 53 healthy subjects (24 men and 29 women, Table 2) were examined to evaluate the differences in each of macular areas between right and left eyes. Finally, 206 right eyes from 206 subjects (88 men and 118 women, Table 3) were examined to evaluate choriocapillaris vasculature changes caused by systemic hypertension.

**Table 1:** Subject characteristics in quantification of normative choriocapillaris vasculature and factors.

	All	Male	Female	P*
Number (eyes)	308	147	161	
Mean age (years)	61.5 ± 10.1	62.8 ± 9.7	60.6 ± 10.4	0.073
Mean visual acuity (logMAR)	−0.03 ± 0.10	−0.04 ± 0.10	−0.02 ± 0.10	0.059
Mean IOP (mmHg)	13.1 ± 2.6	12.9 ± 2.5	13.3 ± 2.6	0.16
Mean AL (mm)	23.99 ± 1.27	24.18 ± 1.14	23.83 ± 1.34	0.0036
Mean VD (%)	42.9 ± 1.1	42.8 ± 1.1	42.9 ± 1.1	0.36
Mean VL (mm/mm^2^)	22.6 ± 1.1	22.7 ± 1.1	22.6 ± 1.1	0.26
Mean VDI (µm)	19.0 ± 0.7	18.9 ± 0.6	19.0 ± 0.7	0.22

*Analyzed by Mann–Whitney *U* test between males and females. logMAR: logarithm of the minimum angle resolution, IOP: intraocular pressure, AL: axial length, VD: vessel density, VL: vessel length, VDI: vessel diameter index.

**Table 2:** Subject characteristics in asymmetric comparison.

	All	Male	Female	P*
Number (person)	53	24	29	
Mean age (years old)	61.1 ± 8.6	64.0 ± 9.2	58.8 ± 7.5	0.067
Mean visual acuity (logMAR)				
Right eye	0.01 ± 0.12	0.01 ± 0.12	0.01 ± 0.13	0.97
Left eye	0.00 ± 0.12	−0.03 ± 0.12	0.01 ± 0.12	0.31
Mean intraocular pressure (mmHg)				
Right eye	12.8 ± 2.6	12.2 ± 2.4	13.3 ± 2.7	0.14
Left eye	13.4 ± 2.9	12.8 ± 2.8	14.0 ± 3.0	0.22
Mean axial length (mm)				
Right eye	24.11 ± 1.25	24.50 ± 1.41	23.79 ± 1.02	0.036
Left eye	24.00 ± 1.12	24.30 ± 1.09	23.72 ± 1.09	0.064

*Analyzed with Mann–Whitney *U* test between males and females.

**Table 3:** Subjects characteristics in measurement of early hypertensive choriocapillaris vasculature change.

KWB	G0	G1	G2	P
Number	159	35	12	
Male/Female	68/91	16/19	4/8	
Systemic condition				
Mean age (years)	61.7 ± 8.5	66.4 ± 9.9	66.2 ± 10.7	0.027
Mean BMI (kg/m^2^)	23.5 ± 3.4	23.2 ± 3.6	23.9 ± 3.6	0.46
Mean SBP (mmHg)	135 ± 19	144 ± 22	153 ± 21	0.006
Mean DBP (mmHg)	80 ± 12	83 ± 14	89 ± 19	0.028
Mean BP (mmHg)	99 ± 14	104 ± 16	110 ± 19	0.004
Ocular condition				
Mean visual acuity (logMAR)	−0.04 ± 0.10	0.00 ± 0.10	0.02 ± 0.11	0.14
Mean IOP (mmHg)	13.1 ± 2.3	12.8 ± 2.9	12.5 ± 1.6	0.33
Mean AL (mm)	23.81 ± 0.91	23.68 ± 1.00	23.96 ± 1.46	0.34
Medication				
Antihypertensive (number, %)	47 (29.6%)	20 (57.1%)	6 (50%)	
Hypoglycemic (number, %)	5 (3.1%)	1 (2.9%)	1 (8.3%)	
Antihyperlipidemic (number, %)	44 (27.7%)	13 (37.1%)	3 (25%)	

Statistically analyzed by non-repeated measures ANOVA. KWB: Keith–Wagener–Barker classification, BMI: body mass index, SBP: systolic blood pressure, DBP: diastolic blood pressure, BP: blood pressure, IOP: intraocular pressure, AL: axial length.

### Quantification of normative choriocapillaris vasculature and affecting factors

**Figure 1:**
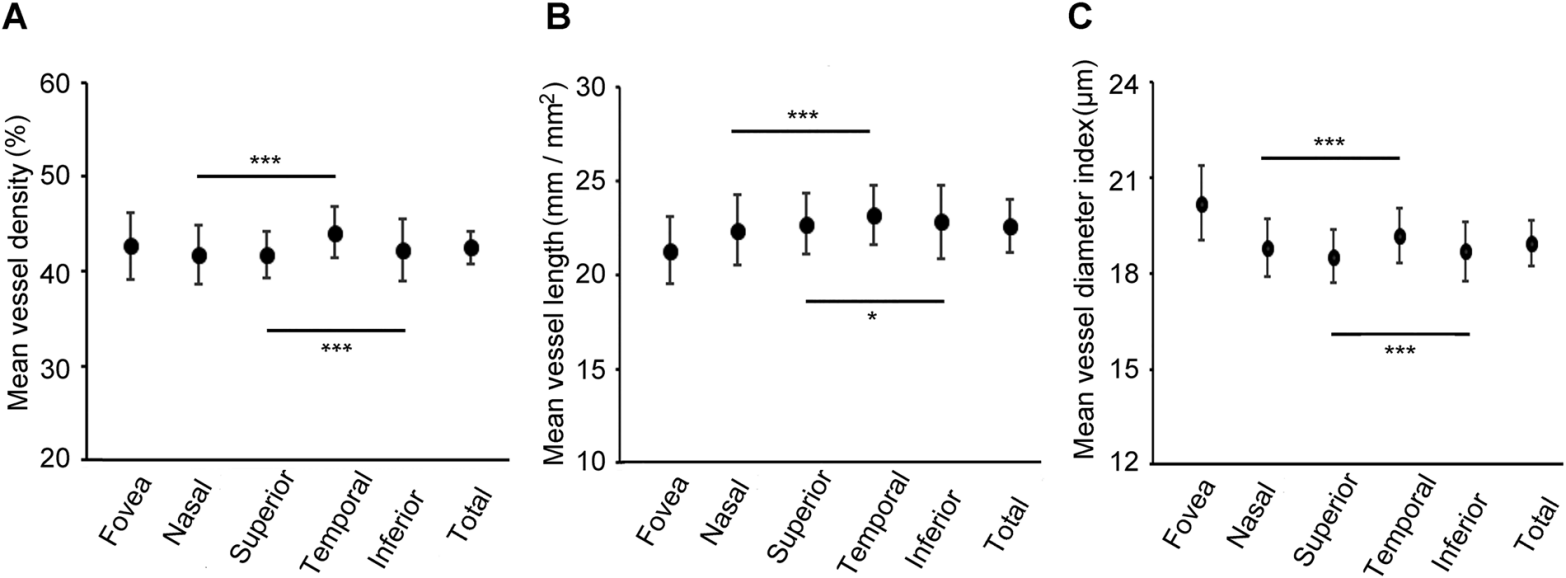
Outcomes of vessel density, vessel length, and vessel diameter index in both eyes. (**A**) The mean vessel density in each sector of all, male, and female subjects. (**B**) The mean vessel length in each sector of all, male, and female subjects. (**C**) The mean vessel diameter index in each sector of all, male, and female subjects. *P < 0.05, **P < 0.01.

We examined normative macula choriocapillaris VD, VL, and VDI in healthy right eyes to detect whether any ocular/systemic factor affects choriocapillaris vasculature. There was a significant difference in AL between males and females (*P* = 0.0036), but no significant differences were detected between males and females in other parameters including VD, VL, and VDI. Comparisons of VD, VL, and VDI between the nasal, temporal, superior, and inferior areas are shown in Fig. 1 and Supplementary Material 1. VD, VL, and VDI were significantly greater in the temporal than in the nasal area (all *P* < 0.001) and significantly greater in the inferior than in the superior area (*P* < 0.001, *P* = 0.011, *P* < 0.001, respectively).

The correlations among VD, VL, VDI, and other parameters (age, IOP, and AL) are shown in Table 4. Age showed negative correlations with VDs and VLs in the fovea, nasal, superior, temporal, and total areas, whereas AL showed negative correlations with VL in the superior, temporal, inferior, and total areas and positive correlations with VDIs in the nasal, superior, temporal, inferior, and total areas. Multiple linear regression analysis suspected the correlations between VD, VL, VDI, and other parameters (Table 5). Age showed negative correlations with VDs and VLs in all areas except the inferior area. IOP showed positive correlations with VLs and negative correlations with VDIs in all areas. AL showed positive correlations with VDIs in all areas except the fovea area. VD in the fovea area was affected by age only, but the effect was weak.

**Table 4:** Correlations of normative choriocapillaris vasculature with other parameters.

	Fovea		Nasal		Superior		Temporal		Inferior		Total	
	P	r	P	r	P	R	P	r	P	r	P	r
Age												
VD	<0.001	–0.19	<0.001	−0.20	0.040	−0.10	<0.001	−0.14	0.26		<0.001	−0.24
VL	0.004	−0.16	0.016	−0.13	0.002	−0.16	0.014	−0.18	0.18		0.004	−0.16
VDI	0.35		0.28		0.06		0.10		0.34		0.19	
IOP												
VD	0.48		0.09		0.34		0.40		0.43		0.47	
VL	0.33		0.13		0.37		0.38		0.28		0.40	
VDI	0.06		0.13		0.10		0.05		0.17		0.05	
AL												
VD	0.06		0.42		0.08		0.34		0.05		0.07	
VL	0.08		0.06		0.016	−0.12	0.010	−0.13	<0.001	−0.23	0.002	−0.16
VDI	0.50		0.003	0.16	0.049	0.10	<0.001	0.25	<0.001	0.22	<0.001	0.22

IOP: intraocular pressure, AL: axial length, VD: vessel density, VL: vessel length, VDI: vessel diameter index.

**Table 5:** Relating factors of normative choriocapillaris vasculature.

	Fovea		Nasal		Superior		Temporal		Inferior		Total	
	β	P	β	P	Β	P	β	P	β	P	β	P
VD												
Sex		0.07		0.86		0.15		0.99		0.79		0.59
Age	−0.060	0.002	−0.036	0.015	−0.02	0.012	−0.034	0.018		0.79	−0.027	<0.001
IOP		0.93		0.16		0.99		0.77		0.84		0.52
AL		0.06		0.13		0.08		0.77	−0.17	0.043		0.10
VL												
Sex		0.34		0.07		0.08		0.62		0.29		0.11
Age	−0.30	0.002	−0.18	0.046	−0.24	0.005	−0.30	<0.001		0.73	−0.19	0.007
IOP	0.70	0.045	0.89	0.010	0.42	0.016	0.44	0.015	0.59	0.048	0.60	0.016
AL		0.035	−2.35	0.002	−1.88	0.004	−2.41	<0.001	−2.77	<0.001	−2.27	<0.001
VDI												
Sex		0.07		0.12		0.99		0.46		0.26		0.06
Age		0.92		0.73		0.050		0.074		0.40		0.48
IOP	−0.63	0.013	−0.40	0.043	−0.34	0.048	−0.42	0.023	−0.45	0.031	−0.42	0.006
AL		0.82	1.17	0.006	0.85	0.034	2.14	<0.001	1.48	<0.001	1.30	<0.001

IOP: intraocular pressure, AL: axial length, VD: vessel density, VL: vessel length, VDI: vessel diameter index.

### Asymmetry of normative right and left eyes

**Figure 2:**
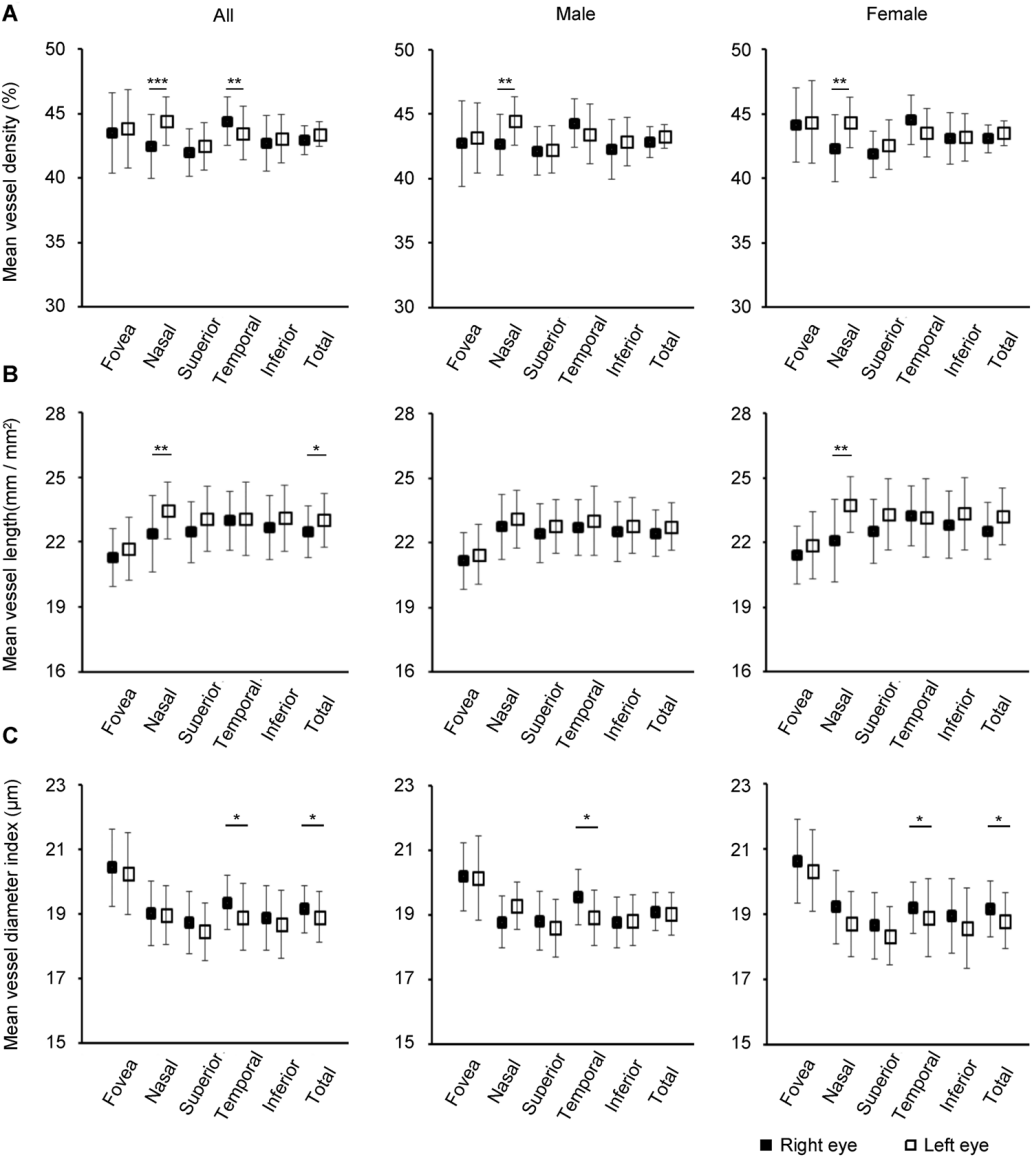
Vessel density, vessel length, and vessel diameter index in the choriocapillaris layer. (**A**) The mean vessel density in each sector. (**B**) The mean vessel length in each sector. (**C**) The mean vessel diameter index in each sector. *P < 0.05, ***P < 0.001.

Ruiz-Medrano *et al*. reported an asymmetry in normative macular choroidal thickness with a trend for right eyes to having a thicker macular nasal choroid layer than left eyes^40^, although no difference in choriocapillaris vasculature has been reported between the right and left eyes. We hypothesized that there would be significant differences in normative macular choriocapillaris vasculature between right and left eyes. We assessed the normative choriocapillaris vasculature in the fovea, nasal, superior, temporal, and inferior areas using OCTA and evaluated the differences detected in each of those areas between right and left eyes (Fig. 2 and Supplementary Materials 2, 3 and 4). VD in the nasal area of right eyes was significantly larger than in left eyes in all subjects (*P* < 0.001), male subjects (*P* = 0.0066), and female subjects (*P* = 0.0053). VD in the temporal area of left eyes was significantly larger than in right eyes in all subjects (*P* = 0.0096), male subjects (*P* = 0.045), and female subjects (*P* = 0.046). VL in the nasal area of left eyes was significantly longer than in right eyes in all subjects (*P* = 0.0017) and female subjects (*P* = 0.0017). VL in the total area of left eyes was significantly longer than in right eyes in all subjects (*P* = 0.031). VDI in the temporal area of right eyes was significantly larger than in left eyes in all subjects (*P* = 0.032), male subjects (*P* = 0.022), and female subjects (*P* = 0.046). VDI in the total area of right eyes was significantly larger than in left eyes in all subjects (*P* = 0.004) and female subjects (*P* = 0.003).

Subsequently, we detected whether any parameter (age, sex, IOP, and laterality) affects VD, VL, and VDI using multiple linear regression analysis. The outcomes are shown in Table 6. Laterality had correlations with VD in the nasal and temporal areas (*P* < 0.001 and *P* = 0.009), VL in the nasal area (*P* = 0.001), and VDI in the temporal area (*P* = 0.03). As for the significant differences in normative choriocapillaris vasculature between right and left eyes, only data pertaining to right eyes were used in the subsequent analysis.

**Table 6:** Correlations between choriocapillaris vasculature and other factors.

	Age	Sex	IOP	Laterality
Vessel density				
Fovea	0.79	0.11	0.37	0.65
Nasal	0.81	0.37	0.52	<0.001
Superior	0.91	0.91	0.07	0.12
Temporal	0.19	0.67	0.28	0.009
Inferior	0.93	0.58	0.22	0.65
Total	0.56	0.85	0.40	0.05
Vessel length				
Fovea	0.74	0.68	0.07	0.25
Nasal	0.65	0.60	0.28	<0.001
Superior	0.78	0.67	0.96	0.06
Temporal	0.83	0.62	0.32	0.95
Inferior	0.03	0.29	0.22	0.28
Total	0.51	0.66	0.26	0.06
Vessel diameter index				
Fovea	0.56	0.17	0.38	0.52
Nasal	0.32	0.92	0.42	0.99
Superior	0.84	0.73	0.10	0.27
Temporal	0.09	0.29	0.67	0.03
Inferior	0.01	0.52	0.76	0.42
Total	0.17	0.71	0.28	0.20

### Measurement of early changes in hypertensive choriocapillaris vasculature

**Figure 3:**
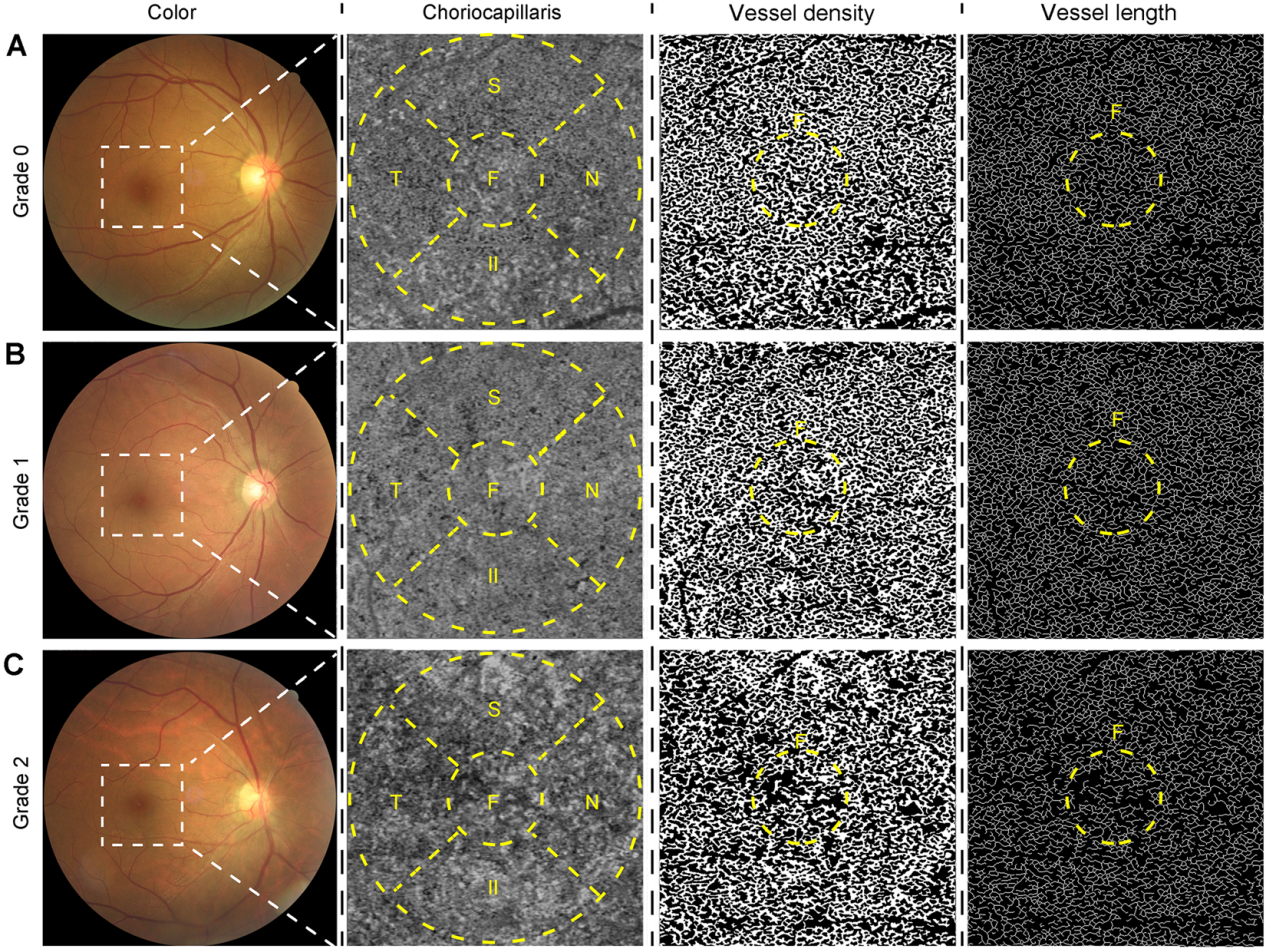
Representative cases of grades 0, 1, and 2 choriocapillaris vasculature. Color photos, choriocapillaris images, vessel density images, and vessel length images of grades 0 (**A**), 1 (**B**), and 2 (**C**). White dashes imply a 3 × 3 mm^2^ area, and these measurements are performed for each Early Treatment Diabetic Retinopathy Study sector (yellow dashes). F, N, S, T, and I indicate the fovea, nasal, superior, temporal, and inferior areas, respectively. Vessel density and vessel length were changed according to the hypertensive progression.

**Figure 4:**
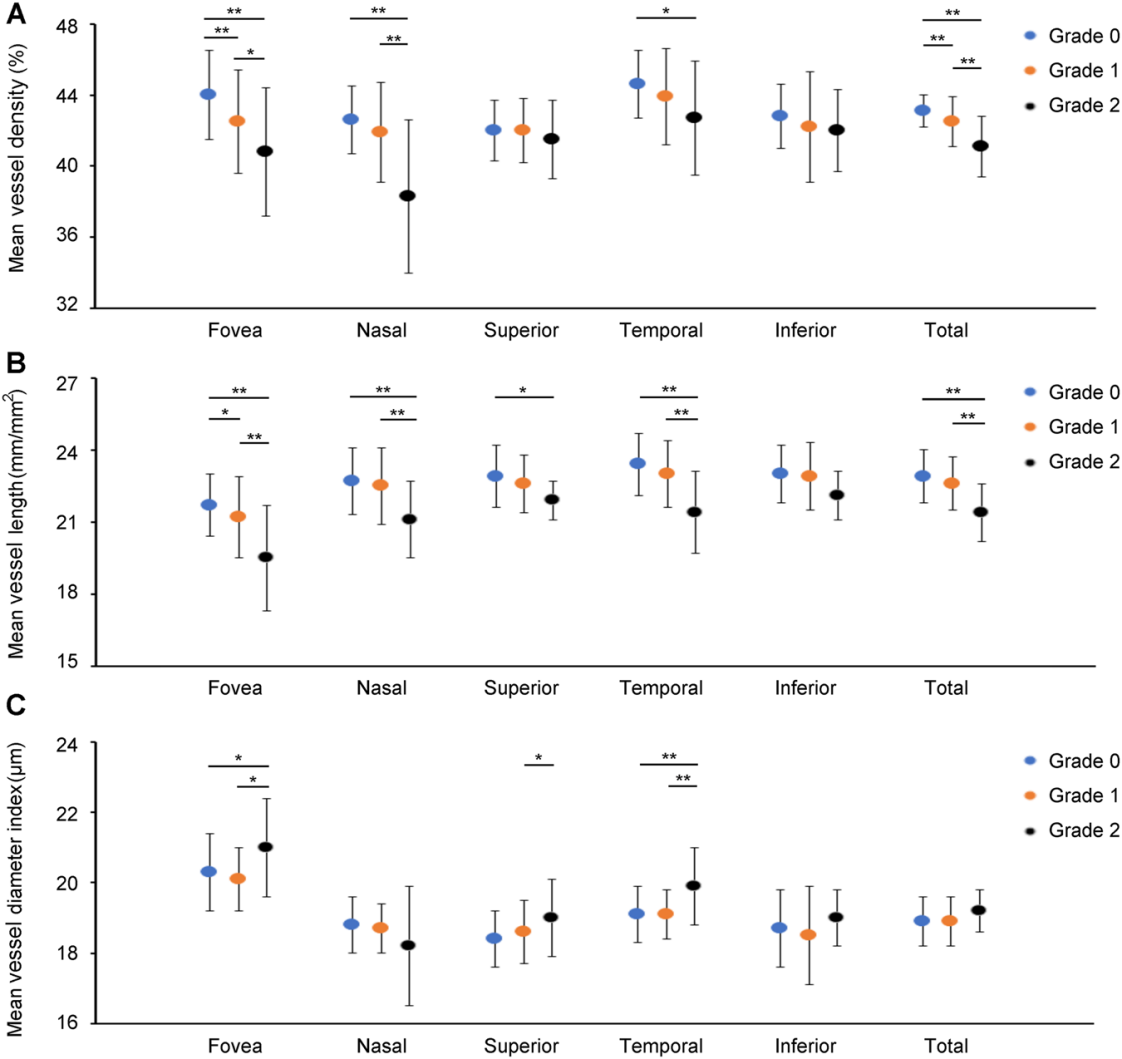
Outcomes of choriocapillaris vasculature comparison among the three groups. (**A**) There were significant differences in the foveal, nasal, temporal, and total area vessel density among the three groups and significant differences in the foveal and total areas among the three grades. (**B**) There were significant differences in vessel length among the three groups in the foveal, nasal, superior, temporal, inferior, and total areas and significant differences in the foveal area among the three groups. (**C**) There were significant differences in vessel diameter index among the three groups in the foveal, superior, and temporal areas. SNK did not detect any significant difference in all areas among the three groups. *P < 0.05, **P < 0.01.

Retinal vasculature and choroidal vasculature are also affected by systemic hypertension, although OCTA cannot distinguish between the small-vessel and large-vessel layers of the retinal and choroidal vasculatures and might generate an overlapped microvascular image. In addition, retinal vasculature can be changed by several retinal diseases, that is, diabetic retinopathy^21–23^, retinal vascular occlusion^24^, and epiretinal membrane disorders^18^; therefore, the retinal vasculature is expected to suffer changes that make the estimation of systemic hypertensive vascular changes difficult. In addition, the choriocapillaris layer contains several capillary vessels^30^, and it is tightly arranged in the posterior pole. On the basis of these features, we hypothesized that choriocapillaris vasculature would be affected by systemic hypertensive vascular changes. Later on, we chose to measure the choriocapillaris vasculature in eyes with and without systemic hypertension and compared the outcomes with those that were based on the KWB classification. In a total of 206 eyes from 206 subjects (88 men and 118 women), 159 eyes were classified as grade 0 (G0); 35 eyes as grade 1 (G1); and 12 eyes as grade 2 (G2). Of all the parameters, only the mean values of age (*P* = 0.027), systolic blood pressure (SBP) (*P* = 0.006), diastolic blood pressure (DBP) (*P* = 0.028), and mean BP (*P* = 0.004) differed significantly among the groups. Representative cases showing a remarkable diminishment of the choriocapillaris vasculature according to the KWB grading and their outcomes are shown in Figs 3 and 4 and Supplementary Material 5. The Student–Newman–Keulis (SNK) test detected significant differences in VD of the fovea and overall areas (fovea: G0/G1 *P* < 0.01, G0/G2 *P* < 0.01, G1/G2 *P* < 0.05; total: G0/G1 *P* < 0.01, G0/G2 *P* < 0.01, G1/G2 *P* < 0.01) and in VL of the fovea area among the three groups (G0/G1, *P* < 0.05, G0/G2 *P* < 0.01, G1/G2 *P* < 0.01), although the SNK test did not detect any significant difference in VDI of all areas among the groups. Multiple linear regression analysis suspected the correlations between VD, VL, VDI, and systemic factors (sex, age, body mass index [BMI], SBP, and DBP) and ocular factors (best-corrected visual acuity [BCVA], IOP, KWB grade, and AL). The outcomes are shown in Table 7 (VD), Table 8 (VL), and Table 9 (VDI). Significant correlations were only detected between KWB grade and VD of the foveal VD (*P* < 0.001, β = −1.62), nasal (*P* < 0.001, β = −1.24), temporal (*P* = 0.004, β = −0.82), and total areas (*P* < 0.001, β = −0.75), and VL of the foveal area (*P* < 0.001, β = −0.79). VLs in other areas and VDI were significantly correlated with other factors or multiple factors including KWB grade. VD and VL in the fovea area showed negative correlation with KWB grade only. The findings suggested that foveal VD was correlated with chronic systemic hypertensive changes and not only with BP measured at the health check-up. We have concluded that OCTA measurement of the foveal choriocapillaris vasculature might be a preferable method for detecting systemic hypertensive changes because of its objective evaluation and automatic use during health check-ups.

**Table 7:** Factors affecting choriocapillaris vessel density.

	Fovea	Nasal	Superior	Temporal	Inferior	Total
Systemic condition						
Sex	0.874	0.149	0.265	0.146	0.581	0.874
Age	0.232	0.102	0.081	0.078	0.070	0.059
BMI	0.062	0.390	0.909	0.733	0.936	0.782
SBP	0.090	0.360	0.405	0.568	0.986	0.994
DBP	0.067	0.160	0.281	0.512	0.883	0.849
Ocular condition						
Visual acuity	0.976	0.074	0.452	0.369	0.900	0.492
IOP	0.719	0.185	0.796	0.828	0.685	0.475
KWB grading	<0.001 (β = −1.62)	<0.001 (β = −1.24)	0.682	0.004 (β = −0.82)	0.095	<0.001 (β = −0.75)
AL	0.487	0.189	0.229	0.440	0.186	0.873
Medication						
Antihypertensive	0.298	0.155	0.554	0.677	0.923	0.996
Hypoglycemic	0.070	0.490	0.544	0.213	0.309	0.288
Antihyperlipidemic	0.073	0.486	0.479	0.627	0.889	0.457

BMI: body mass index, SBP: systolic blood pressure, DBP: diastolic blood pressure, IOP: intraocular pressure, KWB: Keith–Wagener–Barker classification, AL: axial length.

**Table 8:** Factors affecting choriocapillaris vessel length.

	Fovea	Nasal	Superior	Temporal	Inferior	Total
Systemic condition						
Sex	0.122	0.026 (β = −0.48)	0.223	0.514	0.525	0.228
Age	0.134	0.055	0.022 (β = −0.03)	0.028 (β = −0.03)	0.942	0.040 (β = −0.02)
BMI	0.467	0.280	0.185	0.572	0.265	0.309
SBP	0.755	0.380	0.196	0.949	0.912	0.584
DBP	0.912	0.694	0.192	0.389	0.824	0.453
Ocular condition						
Visual acuity	0.916	0.314	0.499	0.798	0.195	0.363
IOP	0.084	0.049 (β = 0.08)	0.566	0.645	0.362	0.169
KWB grading	<0.001 (β = −0.79)	0.040 (β = −0.39)	0.106	<0.001 (β = −0.69)	0.060	0.001 (β = −0.46)
AL	0.230	0.522	0.030 (β = −0.22)	0.043 (β = −0.22)	0.009 (β = −0.26)	0.027 (β = −0.18)
Medication						
Antihypertensive	0.707	0.693	0.929	0.789	0.721	0.894
Hypoglycemic	0.339	0.622	0.937	0.591	0.602	0.755
Antihyperlipidemic	0.265	0.695	0.476	0.649	0.960	0.720

BMI: body mass index, SBP: systolic blood pressure, DBP: diastolic blood pressure, IOP: intraocular pressure, KWB: Keith–Wagener–Barker classification, AL: axial length.

**Table 9:** Factors affecting choriocapillaris vessel diameter index.

	Fovea	Nasal	Superior	Temporal	Inferior	Total
Systemic condition						
Sex	0.033 (β = 0.36)	0.125	0.600	0.398	0.292	0.110
Age	0.679	0.489	0.243	0.378	0.223	0.204
BMI	0.239	0.608	0.111	0.218	0.281	0.129
SBP	0.103	0.841	0.358	0.370	0.993	0.393
DBP	0.038 (β = −0.02)	0.301	0.490	0.033 (β = −0.02)	0.682	0.323
Ocular condition						
Visual acuity	0.990	0.467	0.970	0.143	0.221	0.529
IOP	0.088	0.243	0.315	0.367	0.588	0.171
KWB grading	0.776	0.035 (β = −0.24)	0.083	0.031 (β = 0.22)	0.640	0.521
AL	0.581	0.021 (β = 0.15)	0.120	0.067	<0.001 (β = 0.29)	0.004 (β = 0.15)
Medication						
Antihypertensive	0.541	0.248	0.626	0.923	0.693	0.832
Hypoglycemic	0.420	0.853	0.590	0.450	0.794	0.732
Antihyperlipidemic	0.493	0.785	0.787	0.984	0.877	0.978

BMI: body mass index, SBP: systolic blood pressure, DBP: diastolic blood pressure, IOP: intraocular pressure, KWB: Keith–Wagener–Barker classification, AL: axial length.

## Discussion

In the present study, we measured choriocapillaris vasculature in healthy subjects and systemic hypertensive subjects using OCTA, and the results indicated three points. Firstly, there were significant differences in normative choriocapillaris vascular profile between right and left eyes. Studying choriocapillaris vasculature in both eyes may be problematic in terms of accuracy. Secondly, many factors such as age, IOP, and AL affect normative choriocapillaris vascular profile. Foveal VD was correlated with age only, and the effect was small. Thirdly, foveal VD was strongly correlated with KWB grade, but not with other systemic and ocular factors, that is, age, sex, BP measured at the check-up, or medication. OCTA measurement for foveal choriocapillaris vasculature might provide the advantage of evaluating the progression of systemic hypertension.

Previous studies have reported differences between right and left eyes in normative choroidal thickness based on OCT (optical coherence tomography) findings and the assessment of choriocapillaris vasculature in the total 3 × 3 mm^2^ area with OCTA^40,41^. Ruiz-Medrano *et al*. reported an asymmetry in normative macular choroidal thickness with a trend for right eyes to having a thicker macular nasal choroid layer than left eyes^40^, although no difference in choriocapillaris vasculature has been reported between the right and left eyes. The present study is the first to compare normative choriocapillaris vasculature between right and left eyes using OCTA. VD and VL in the nasal area were larger and longer in left eyes than in right eyes, and these differences were affected by laterality. The differences had adverse tendencies, as previously described for choroidal thickness^40^. The asymmetry of the macular choroidal thickness is considered to originate in the right common carotid artery lying in the brachiocephalic trunk instead of emerging directly from the aorta and thereby presumably being responsible for a more proximal and direct blood flow to the right carotid^42,43^, although the cause of the differences in choriocapillaris vasculature is unclear. Further studies on choriocapillaris vasculature and circulation are needed to elucidate the cause of these differences.

McLeod *et al*.^44^ showed that VD in the choriocapillaris changes depending on the fundus location, forming a densely packed honeycomb structure at the central fovea, whereas in the temporal area, a more lobular and less dense capillary structure can be identified, and in the nasal area, the capillary structure becomes even less dense with larger lobules. The authors indicated that choriocapillaris vasculature in the temporal area has more vessels and larger VD and VL than in the nasal area. The indication was almost the same as in the present study, and the results proved the reliability of OCTA for measuring choriocapillaris vasculature. Choriocapillaris vasculature in the inferior area was also longer and larger in diameter than in the superior area, and VD in the inferior area was greater than that in the superior area. Similarly to a previous choroidal study^45^, a comparison of VD, VL, and VDI in the inferior area with other areas showed that these parameters in the inferior area were not significantly worse than in other areas, contrary to the results of the previous study.

According to histological evaluation, choriocapillaris thickness decreases with age^46^. However, previous OCT examinations detected that the density of the retinal and choriocapillaris network in the central and pericentral region is significantly associated with sex^47^, but not age or AL^12,48,49^. According to swept-source OCT, which enables a precise qualitative and quantitative characterization of the choriocapillaris vasculature, detected that only choriocapillaris thickness is thinner in older eyes^50^. In the present study, VD and VL in the fovea, nasal, superior, temporal, and total areas had a tendency to decrease age dependently, as reported in previous studies. In particular, normative foveal VD was correlated with age only, but the efficiency was small (Table 5). In eyes with or without systemic hypertension, foveal VD was negatively correlated with KWB grade only, ant the efficiency was larger than that of age (Table 7). Spaide *et al*.^26^ reported that blood flow in the macular choriocapillaris vasculature was decreased in the eyes of elderly individuals and those with systemic hypertension, and the indication was almost the same as in the present study. In addition, it was not correlated with SBP and DBP. It is possible that BP measured at the health check-up was affected by environmental and mental factors such as room temperature, circadian variation, or white-coat hypertension, whereas OCTA detects foveal choriocapillaris changes caused by chronic systemic hypertension.

In the present study, VD in the fovea, nasal, superior, temporal, and total areas had tendencies to decrease age dependently, although VD in the inferior area were not correlated with age, but with AL dependently (Table 5). There was no significant correlation with sex, and VD and VL in the inferior area were not correlated with age, but with AL. Magnetic resonance imaging analysis of ocular shape in myopic eyes showed that the most protruding part of the globe was along the central sagittal axis and slightly inferior to the central axis^51^. It was concluded that ocular shape in the inferior area is affected by AL more than that in other areas, although the correlation between VD in the inferior area and AL was unknown. Thus, further studies are needed to elucidate these correlations. VDI in the inferior area was negatively correlated with IOP and positively correlated with AL. It was concluded that the IOP presses on the choriocapillaris layer, which includes the vasculature, and the increased choriocapillaris pressure decreases the width of the vessels. In contrast, eye shapes with long AL change the macular area widely^52^; therefore, a longer AL would change the choriocapillaris vasculature widely. Borrelli reported a correlation between choriocapillaris vasculature and sex by comparing healthy pediatric patients (23 males and 29 females)^47^. The present study compared 147 healthy males and 161 females over 39 years and did not detect any correlation between sex and choriocapillaris vasculature. It is possible that choriocapillaris vasculature growth differs by sex, although the differences between adult male and females are not significant.

We measured the choriocapillaris vasculature in eyes with and without systemic hypertension using OCTA and detected differences in the vasculature according to the KWB classification. To our best knowledge, this was the first report suggesting a correlation between choriocapillaris vasculature in the fovea area and systemic hypertensive changes. Spaide *et al*.^26^ reported that blood flow in the macular choriocapillaris vasculature was decreased in the eyes of elderly individuals and those with systemic hypertension. In their study, blood flow was measured in the entire macular area, not in the foveal area alone. In the present study, we measured vasculature according to the Early Treatment Diabetic Retinopathy Study sectors and found that VD and VL in the fovea area were negatively correlated with KWB grade only, but not correlated with SBP and DBP. It is possible that BP measured at the health check-up was affected by environmental and mental factors such as room temperature, circadian variation, or white-coat hypertension, whereas OCTA detects foveal choriocapillaris changes caused by chronic systemic hypertension.

KWB classification was first described in 1939^53^ and implied that the severity of retinopathy was an indicator of overall mortality in hypertensive patients^54,55^. However, the KWB scale has major limitations. Firstly, it is difficult to distinguish between first and second grades. Secondly, it cannot measure hypertensive changes in eyes with retinal disease, and thirdly and most importantly, this classification is based on subjective scaling among clinicians. To resolve these problems, simplified grading scales were developed; however, these grades were also subjective and not automatically calculated^4^. In the present study, we measured systemic hypertensive changes by OCTA that might eliminate these limitations.

As for the first limitation, the present study showed that the differences in foveal VD and VL between G0, G1, and G2, which are the early stages of systemic hypertensive changes, were difficult to distinguish from each other. OCTA was considered capable of detecting microvascular changes caused by early stages of systemic hypertension (Figs 3 and 4).

Regarding the second limitation, retinal vasculature and choroidal vasculature are also affected by systemic hypertension. In eyes with retinal diseases^16,17^, such as diabetic retinopathy^21–23^, retinal vascular occlusion^24^, and epiretinal membrane disorders^18^, the retinal vasculature is expected to show changes that make the estimation of systemic hypertensive vascular changes difficult. OCTA cannot distinguish between the small-vessel and large-vessel layers of the choroidal vasculature and might generate an overlapped microvascular image. On the basis of these features, we chose to measure the choriocapillaris vasculature in this present study and were able to detect systemic hypertensive changes.

To address the third limitation, we measured the microvasculature using a Cirrus HD-5000^TM^ OCTA machine, which can distinguish between the choriocapillaris vasculature images in the retina and the choroid. The bundled Angioplex^TM^ software can automatically calculate VD and VL^37^. OCTA is a non-invasive, objective measurement that describes vascular changes as numbers. The machine and the analysis methods have been used in many studies^26,56,57^, and other OCTA machines are capable of visualizing and analyzing choriocapillaris vasculature. Foveal choriocapillaris vasculature could be measured and analyzed by OCTA machines^28,58^, and a new objective classification could be implemented using any OCTA machine.

In conclusion, we measured normative VD, VL, and VDI of the choriocapillaris vasculature and the differences in vasculature between the right and left eyes by OCTA. In addition, OCTA for foveal choriocapillaris measurement might provide the advantage of evaluating the progression of systemic hypertension. OCTA might be a novel objective method to evaluate the progression of systemic hypertension from an early stage. Because of the advantages of being an objective and automatic calculation method, OCTA might take the place of the KWB classification in the evaluation of systemic hypertension during health check-ups.

## Methods

### Setting and participants

The subjects were healthy volunteers who attended a basic health check-up supported by the local government in 2016. This check-up (the Yakumo study) was conducted in the town of Yakumo in a rural area of southern Hokkaido, Japan. Subjects were excluded eligible if they met any of the following criteria: (1) BVCA > 20/30, (2) AL > 23 or <27 mm, (3) no history of corneal disease, (4) no history of intraocular disease, (5) no history of diabetes mellitus, (6) no history of systemic diseases that might affect microvascular circulation, and (7) agreed to undergo OCTA measurement in the Yakumo health check-up. The absence of macular disease was verified by OCT (RS-3000, Nidek, Gamagori, Aichi, Japan) and color fundus photos (Topcon TRC-NW8, Tokyo, Japan). Eyes with a signal strength of OCTA ≥ 6/10 were included, whereas those with a signal strength of <6/10 and with poor quality images on OCTA or OCT because of eye movement or media opacities were excluded.

All subjects aged 39 years or older underwent assessment of BMI (weight in kg/height in m^2^), SBP, DBP, and mean BP. BP was measured using an automated oscillometric monitor (Omron HBP-9021, Omron Healthcare, Kyoto, Japan). Omron RS6 (HEM-6221-E) is an automatic device for the self-measurement of BP at the wrist level using the oscillometric method. The wrist cuff circumference is suitable for wrist circumferences of 13.5–21.5 cm. The device has a digital liquid crystal display screen that displays the measured BP and pulse rate. The unit measures pressures from 0 to 299 mmHg and pulses from 40 to 180 beats/min. We measured in triplicate, and the average was used. Intake of antihypertensive, hypoglycemic, and/or antihyperlipidemic drugs was noted, and those without any history of intraocular or systemic circulatory disease were included in the study. This cross-sectional, non-interventional study was approved by the institutional review board of Nagoya University Hospital and was conducted in accordance with the principles of the Declaration of Helsinki. Informed consent was obtained from all study subjects before any procedures were carried out.

### Best-corrected visual acuity and axial length

BCVA was measured by a standard Japanese visual acuity chart. The decimal BCVA was converted to the logarithm of the minimum angle of resolution (logMAR) for statistical analysis^59,60^. AL was measured with an IOL master model 500 (Carl Zeiss, Germany). AL was calculated 10 times, and the average reading was used.

### Optical coherence tomography angiography

OCTA (Cirrus Angiography ZEISS Angioplex™, Carl Zeiss Meditec, Inc., Dublin, USA) generates high-resolution, three-dimensional maps of the retinal and choroidal microvasculature while retaining all the capabilities of the CIRRUS™ HD-OCT Model 5000 instrument. In this system, OCTA measurement was made possible by increasing the scanning rate to 68,000 A scans per second and by introducing an improved retinal tracking software technology (FastTrac™)^61^. The generation of en face microvascular flow images with the Angioplex™ OCT with projection artifact removal function involved the use of an algorithm known as the OCT microangiographic complex, which accounted for differences in both phase and intensity information contained within sequential B scans performed at the same position. The current scanning patterns for en face angiographic visualization included a 3 × 3 mm scan pattern on the choriocapillaris.

### Measurement of the choriocapillaris vasculature

From the obtained OCTA images, the choriocapillaris layer was imaged starting from the inferior area 29 µm below RPE to 49 µm below RPE, as previous described^62^. The image segmentation and analysis were performed automatically by the machine-bundled software and then checked by three independent and well-trained observers (K. T., Y. I., and T. A.) who were blinded to the clinical data. Motion correction was performed by the registration of two orthogonally captured imaging volumes. Macular area in a 3 × 3 mm^2^ circle was automatically classified at the fovea and at each of the other four sites (nasal, superior, temporal, and inferior) as defined by the ETDRS grid, as previously described^40^. VD and VL in each sector were automatically measured by the machine-bundled software. VD was shown as percent units, as previously described, and VL was shown as mm/mm^2^ units. VDI was shown as µm units.

### Keith–Wagener–Barker classification

The right eye of each subject was photographed using a 45° digital non-mydriatic retinal camera after adaptation in the dark room; retinal images centered on the fovea were obtained. Subsequently, these photographs were randomly graded using the KWB classification system^63^ by three independent and well-trained observers (K. T., Y. I., and K. K.) who were blinded to the clinical data. The intraobserver level of agreement for both hypertensive grading systems was determined through a second grading by one of the three clinicians (Y. I.).

### Statistical analyses

Data are shown as means ± standard deviation. The Mann–Whitney *U* test was used to analyze differences between data pertaining to nasal, superior, temporal, and inferior areas in right and left eyes in all subjects, male subjects, and female subjects and differences between data from male and female subjects, temporal and nasal areas, and superior and inferior areas from right eyes in normative subjects. Spearman’s correlation was used to analyze the statistical correlation between the two groups. Non-repeated measure of analysis of variance was used to test for differences in outcomes among the three grades. The SNK test was used to analyze further the differences among the three KWB grades. Multiple linear regression analysis was performed to evaluate the relationships between VD, VL, and VDI and ocular factors such as sex, IOP, AL, and KWB classification. *P* values < 0.05 were considered to indicate statistical significance.

## Electronic supplementary material

Supplementary table S1, S2, S3, S4, and S5
