# Supplementary material for: Novel Classification of Early-stage Systemic Hypertensive Changes in Human Retina Based on OCTA Measurement of Choriocapillaris
Source: Sci Rep. 2018 Oct 11;8:15163. doi: 10.1038/s41598-018-33580-y (PMC6181956; doi:10.1038/s41598-018-33580-y)
Supplement: Supplementary file 1 — Supplementary table S1, S2, S3, S4, and S5 [file 41598_2018_33580_MOESM1_ESM.pdf]

Novel Classification of Early-stage Systemic Hypertensive Changes Based on OCTA Measurement of Choriocapillaris

Kei Takayama, Hiroki Kaneko, Yasuki Ito, Keiko Kataoka, Takeshi Iwase, Tetsuhiro Yasuma, Toshiyuki Matsuura, Taichi Tsunekawa, Hideyuki Shimizu, Ayana Suzumura, Eimei Ra, Tomohiko Akahori, Hiroko Terasaki

Suppprementary Table S1. Normatie choriocapillaris vasculature in each ETDRS sector of right eyes

	Fovea	Nasal	Superior	Temporal	Inferior	Total
VD (%)	43.1 ± 3.0	42.1 ± 2.3	42.0 ± 1.8	44.0 ± 2.2	42.8 ± 1.9	42.9 ± 1.1
VL (mm / mm^2)	21.3 ± 1.5	22.4 ± 1.5	22.7 ± 1.3	23.2 ± 1.4	22.9 ± 1.3	22.4 ± 1.1
VDI (µm)	20.2 ± 1.1	18.8 ± 0.9	18.5 ± 0.8	19.2 ± 0.8	18.7 ± 0.9	19.0 ± 0.7

VD: vessel density, VL: vessel length, VDI: vessel diameter index

Suppramentary Table S2. Normative vessel density in right and left eye

	Fovea	Nasal	Superior	Temporal	Inferior	Total
All						
Right eye	43.4 ± 3.1	42.4 ± 2.5	42.0 ± 1.8	44.4 ± 1.9	42.7 ± 2.1	43.0 ± 2.1
Left eye	43.8 ± 3.0	44.4 ± 1.9	42.5 ± 1.9	43.5 ± 1.9	43.0 ± 1.9	43.4 ± 1.9
P value	0.47	<0.001	0.25	0.0096	0.39	0.068
Male						
Right eye	42.7 ± 3.3	42.6 ± 2.4	42.1 ± 1.9	44.3 ± 1.9	42.3 ± 2.3	42.9 ± 1.2
Left eye	43.2 ± 2.7	44.5 ± 1.9	42.3 ± 1.8	43.4 ± 2.3	42.9 ± 1.9	43.2 ± 0.9
P value	0.43	0.0066	0.81	0.045	0.37	0.26
Female						
Right eye	44.1 ± 2.9	42.3 ± 2.6	41.9 ± 1.8	44.5 ± 1.9	43.1 ± 2.0	43.1 ± 1.1
Left eye	44.4 ± 3.2	43.2 ± 1.9	42.6 ± 1.9	43.5 ± 1.9	43.2 ± 1.9	43.5 ± 1.0
P value	0.73	0.0053	0.16	0.046	0.62	0.16

Statistically analyzed by Mann-Whitney *U* test

Suppprementary Table S3. Normative vessel length in right and left eye and classification into male and female.

	Fovea	Nasal	Superior	Temporal	Inferior	Total
All						
Right eye	21.3 ± 1.3	22.4 ± 1.8	22.5 ± 1.4	23.0 ± 1.4	22.7 ± 1.5	22.5 ± 1.2
Left eye	21.7 ± 1.5	23.5 ± 1.3	23.1 ± 1.7	23.1 ± 1.5	23.0 ± 1.2	23.0 ± 1.2
P value	0.21	0.0017	0.051	0.73	0.12	0.031
Male						
Right eye	21.1 ± 1.3	22.7 ± 1.5	22.4 ± 1.4	22.7 ± 1.3	22.5 ± 1.4	22.4 ± 1.1
Left eye	21.5 ± 1.4	23.1 ± 1.3	22.8 ± 1.2	23.0 ± 1.6	22.8 ± 1.3	22.7 ± 1.1
P value	0.47	0.33	0.37	0.62	0.37	0.31
Female						
Right eye	21.4 ± 1.4	22.1 ± 1.9	22.5 ± 1.5	23.2 ± 1.4	22.8 ± 1.6	22.5 ± 1.3
Left eye	21.9 ± 1.5	23.8 ± 1.3	23.3 ± 1.7	23.1 ± 1.8	23.3 ± 1.7	23.2 ± 1.3
P value	0.30	0.0017	0.069	0.90	0.21	0.058

Statistically analyzed by Mann-Whitney *U* test

Suppremetary Table S4. Normative vessel diameter index in right and left eye and classification into male and female.

	Fovea	Nasal	Superior	Temporal	Inferior	Total
All						
Right eye	20.4 ± 1.2	19.0 ± 1.0	18.7 ± 1..0	19.3 ± 0.8	18.9 ± 1.0	19.1 ± 0.7
Left eye	20.3 ± 1.3	19.0 ± 0.9	18.5 ± 0.9	18.9 ± 1.0	18.7 ± 1.1	18.9 ± 0.8
P value	0.51	0.81	0.16	0.032	0.22	0.049
Male						
Right eye	20.2 ± 1.1	18.8 ± 0.8	18.7 ± 1..0	19.5 ± 0.9	18.8 ± 0.8	19.1 ± 0.6
Left eye	20.2 ± 1.3	19.3 ± 0.7	18.6 ± 0.9	18.9 ± 0.9	18.8 ± 0.8	19.0 ± 0.7
P value	0.78	0.07	0.47	0.022	0.89	0.57
Female						
Right eye	20.6 ± 1.3	19.2 ± 1.1	18.6 ± 1.0	19.2 ± 0.8	18.9 ± 1.1	19.2 ± 0.8
Left eye	20.3 ± 1.2	18.7 ± 1.0	18.3 ± 0.9	18.9 ± 1.2	18.6 ± 1.2	18.8 ± 0.9
P value	0.57	0.06	0.25	0.046	0.13	0.036

Statistically analyzed by non-repeated measures ANOVA

Suppremementary Table S5. Outcomes of choriocapillaris vasculature in the three groups

	Fovea	Nasal	Superior	Temporal	Inferior	Total
Vessel density						
Grade 0	44.0 ± 2.5	42.6 ± 1.9	42.0 ± 1.7	44.6 ± 1.9	42.8 ± 1.8	43.1 ± 0.9
Grade 1	42.5 ± 2.9	41.9 ± 2.8	42.0 ± 1.8	43.9 ± 2.7	42.2 ± 3.1	42.5 ± 1.4
Grade 2	40.8 ± 3.6	38.3 ± 4.3	41.5 ± 2.2	42.7 ± 3.2	42.0 ± 2.3	41.1 ± 1.7
P value#	<0.001	<0.001	0.29	0.0027	0.066	<0.001
Difference between grade						
Grade0/Grade1	**					**
Grade0/Grade2	**	**		*		**
Grade1/Grade2	*	**				**
Vessel length						
Grade 0	21.7 ± 1.3	22.7 ± 1.4	22.9 ± 1.3	23.4 ± 1.3	23.0 ± 1.2	22.9 ± 1.1
Grade 1	21.2 ± 1.7	22.5 ± 1.6	22.6 ± 1.2	23.0 ± 1.4	22.9 ± 1.4	22.6 ± 1.1
Grade 2	19.5 ± 2.2	21.1 ± 1.6	21.9 ± 0.8	21.4 ± 1.7	22.1 ± 1.0	21.4 ± 1.2
P value#	<0.001	<0.001	0.014	<0.001	0.033	<0.001
Difference between grade						
Grade0/Grade1	*					
Grade0/Grade2	**	**	*	**		**
Grade1/Grade2	**	**		**		**
Vessel diameter index						
Grade 0	20.3 ± 1.1	18.8 ± 0.8	18.4 ± 0.8	19.1 ± 0.8	18.7 ± 1.1	18.9 ± 0.7
Grade 1	20.1 ± 0.9	18.7 ± 0.7	18.6 ± 0.9	19.1 ± 0.7	18.5 ± 1.4	18.9 ± 0.7
Grade 2	21.0 ± 1.4	18.2 ± 1.7	19.0 ± 1.1	19.9 ± 1.1	19.0 ± 0.8	19.2 ± 0.6
P value#	0.027	0.051	0.010	0.001	0.23	0.12
Difference between grade						
Grade0/Grade1						
Grade0/Grade2	*			**		
Grade1/Grade2	*		*	**		

#: analyzed between the 3 groups by non-repeated measures ANOVA, *: P < 0.05 **: P < 0.01 by SNK test.
